# Amplification of potential thermogenetic mechanisms in cetacean brains compared to artiodactyl brains

**DOI:** 10.1038/s41598-021-84762-0

**Published:** 2021-03-09

**Authors:** Paul R. Manger, Nina Patzke, Muhammad A. Spocter, Adhil Bhagwandin, Karl Æ. Karlsson, Mads F. Bertelsen, Abdulaziz N. Alagaili, Nigel C. Bennett, Osama B. Mohammed, Suzana Herculano-Houzel, Patrick R. Hof, Kjell Fuxe

**Affiliations:** 1 School of Anatomical Sciences, University of the Witwatersrand, Johannesburg, South Africa; 2 Department of Anatomy, Des Moines University, Des Moines, IA USA; 3 Biomedical Engineering, Reykjavik University, Reykjavik, Iceland; 4 Centre for Zoo and Wild Animal Health, Copenhagen Zoo, Frederiksberg, Denmark; 5 KSU Mammals Research Chair, Department of Zoology, College of Science, King Saud University, Riyadh, Saudi Arabia; 6 Department of Zoology and Entomology, University of Pretoria, Pretoria, South Africa; 7 Department of Psychology, Department of Biological Sciences, Vanderbilt Brain Institute, Vanderbilt University, Nashville, TN USA; 8 Nash Family Department of Neuroscience and Friedman Brain Institute, Icahn School of Medicine at Mount Sinai, New York, NY USA; 9 Department of Neuroscience, Karolinska Institutet, Stockholm, Sweden; 10 Present Address: Institute for the Advancement of Higher Education, Hokkaido University, Sapporo, Japan; 11 Present Address: Division of Clinical Anatomy and Biological Anthropology, Department of Human Biology, University of Cape Town, Cape Town, South Africa

**Keywords:** Cognitive neuroscience, Evolution, Neuroscience, Physiology, Climate sciences, Ecology, Ocean sciences, Anatomy

## Abstract

To elucidate factors underlying the evolution of large brains in cetaceans, we examined 16 brains from 14 cetartiodactyl species, with immunohistochemical techniques, for evidence of non-shivering thermogenesis. We show that, in comparison to the 11 artiodactyl brains studied (from 11 species), the 5 cetacean brains (from 3 species), exhibit an expanded expression of uncoupling protein 1 (UCP1, UCPs being mitochondrial inner membrane proteins that dissipate the proton gradient to generate heat) in cortical neurons, immunolocalization of UCP4 within a substantial proportion of glia throughout the brain, and an increased density of noradrenergic axonal boutons (noradrenaline functioning to control concentrations of and activate UCPs). Thus, cetacean brains studied possess multiple characteristics indicative of intensified thermogenetic functionality that can be related to their current and historical obligatory aquatic niche. These findings necessitate reassessment of our concepts regarding the reasons for large brain evolution and associated functional capacities in cetaceans.

## Introduction

Cetaceans (whales, dolphins and porpoises) in general have large relative and absolute brain sizes, and are often considered cognitively complex mammals, their large brains apparently evolving in response to social and ecological demands present in their evolutionary history^1–3^; however, alternative views regarding cetacean brain structure, function and evolution have been proposed^4–8^. The multiplicity of atypical features of the cetacean brain compared to other mammals includes a homogeneous cerebral cortex^4^, an atypical allometric relationship between the brain and body, lack of a layer IV in the entire cerebral cortex, low numbers of cortical areas, paucity of cortical columnar and mini-columnar organization of cortical neurons, a comparatively low number of neuronal morphotypes, a thin and volumetrically small cerebral cortex, a low cortical neuronal density, a high glia:neuron index, altered proportions of the neuropil, a greatly reduced size of the prefrontal cortex^6^, a small hippocampus that lacks adult hippocampal neurogenesis^8^, a small corpus callosum^9^, unusual and extensive cortical gyrencephaly^10^, and low cortical neuronal complexity^11^. Combined with their unusual sleep physiology^12,13^, and the finding that cetaceans do not outperform other mammals in behavioural tasks^5,7,14^, these features of the cetacean brain mount a significant challenge to the paradigm that cetaceans possess levels of cognitive complexity that differentiate them from the majority of other mammals.

It has been proposed that the current and historical, ubiquitous environmental pressure of water temperature has led to the evolution of the larger absolute and relative size of the cetacean brain^6^. The mammalian brain is particularly sensitive to changes in temperature, with cortical neurons showing optimal functioning between 36 and 37 °C, significantly decreased activity when brain temperature falls to 33°C, and loss of consciousness at 25–26 °C^15^. Thus, maintenance of brain temperature at levels appropriate for optimal neuronal functioning is an important aspect of mammalian physiology. Experimental evidence shows that exposure of the mammalian body to cold results in major decreases in body temperature but does not necessarily induce changes in brain temperature^16^. In addition, the temperature of the blood in the mammalian internal carotid artery is generally lower than that of the brain and jugular venous blood^17,18^. These studies indicate that the mammalian brain itself produces the heat required for optimal neuronal functioning, independent of thermogenetic mechanisms occurring in the remainder of the body. As there is no skeletal muscle within the mammalian cranial cavity, it is logical to posit that the production of heat by the brain would be through non-shivering thermogenetic mechanisms. Brown fat is a well-established site of non-shivering adaptive thermogenesis, and within brown fat, uncoupling proteins (UCPs) have been explicitly linked to the production of heat through their action on mitochondrial molecular pathways^19,20^. Of the UCP family of proteins, all have been observed in the mammalian brain, but UCPs 1, 3, 4 and 5 are particularly strongly expressed and, amongst the many potential physiological attributes of these proteins in the brain, have been functionally linked to thermogenesis^19,21–23^. In addition, one of the many functions of noradrenaline is to control UCP concentrations and rapidly initiate UCP activity in brown adipocytes, leading to increased thermogenesis^24,25^. Given the presence of UCPs and noradrenaline in the mammalian brain we examined the brains of three species of cetacean and eleven species of the closely related artiodactyls (even-toed ungulates, which combined form the order Cetartiodactyla^26^) to explore the potential cellular basis of the thermogenetic hypothesis of cetacean brain evolution^6^.

## Results

### Amplified UCP1 expression in cetaceans

**Figure 1:**
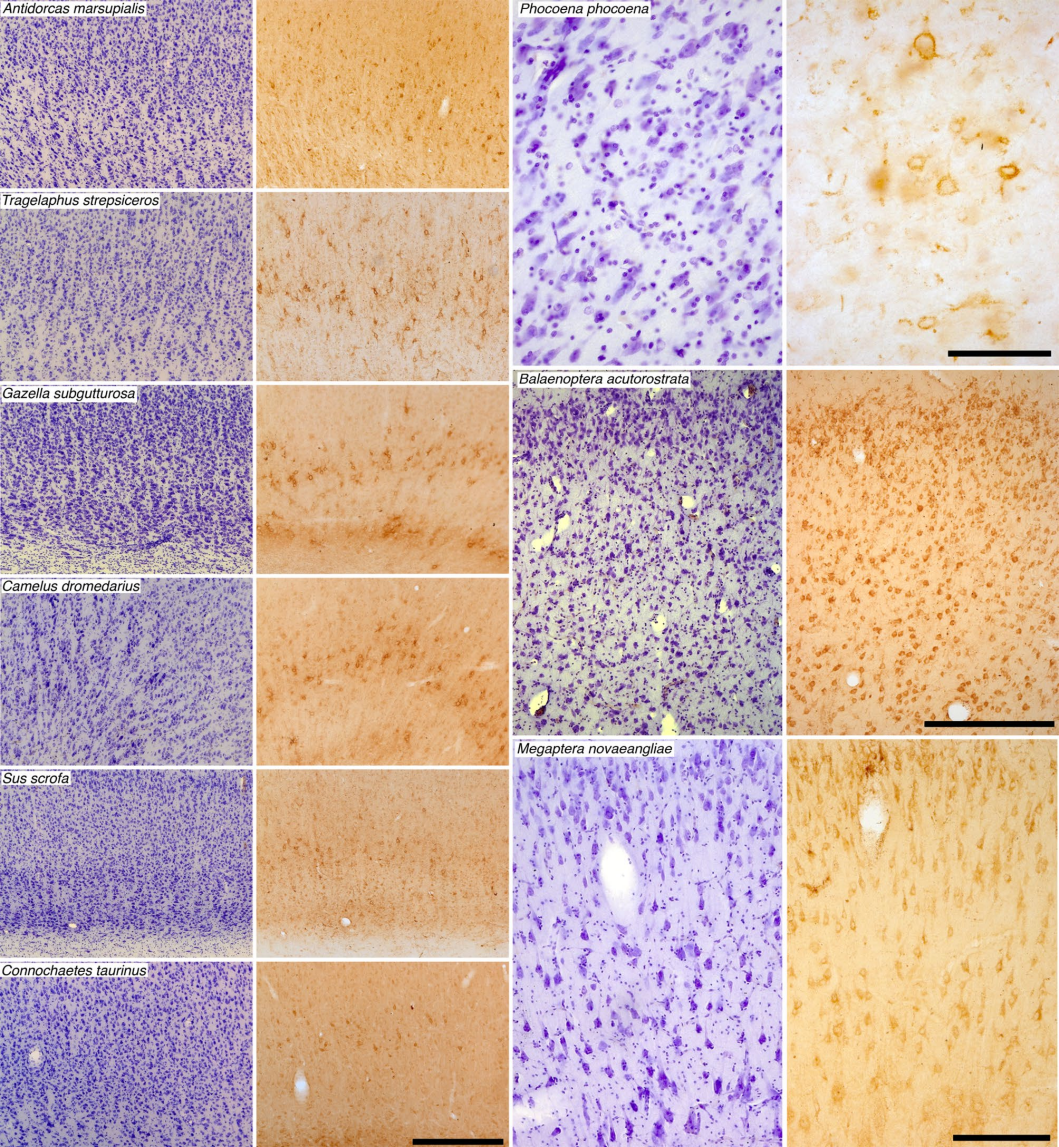
UCP1 immunostaining in cetartiodactyl cerebral cortex. Photomicrographs of Nissl stained (purple colored images) and UCP1-immunostained (brown colored images) cortical sections in a range of artiodactyl (two left columns) and cetacean species (two right columns). Note in all cases the presence of UCP1-immunostained cortical neurons, but in the artiodactyls these are limited to the lower layers of the cortex, while almost all cortical neurons from all layers are immunopositive in the cetaceans. Scale bar in the UCP1-immunostained section of *Connochaetes taurinus* equals 500 µm and applies to all artiodactyl images. Scale bar in the UCP1-immunostained section of *Phocoena phocoena* equals 100 µm and applies to both images. Scale bar in the UCP1-immunostained section of *Balaenoptera acutorostrata* equals 500 µm and applies to both images. Scale bar in the UCP1-immunostained stained section of *Megaptera novaeangliae* equals 250 µm and applies to both images.

**Figure 2:**
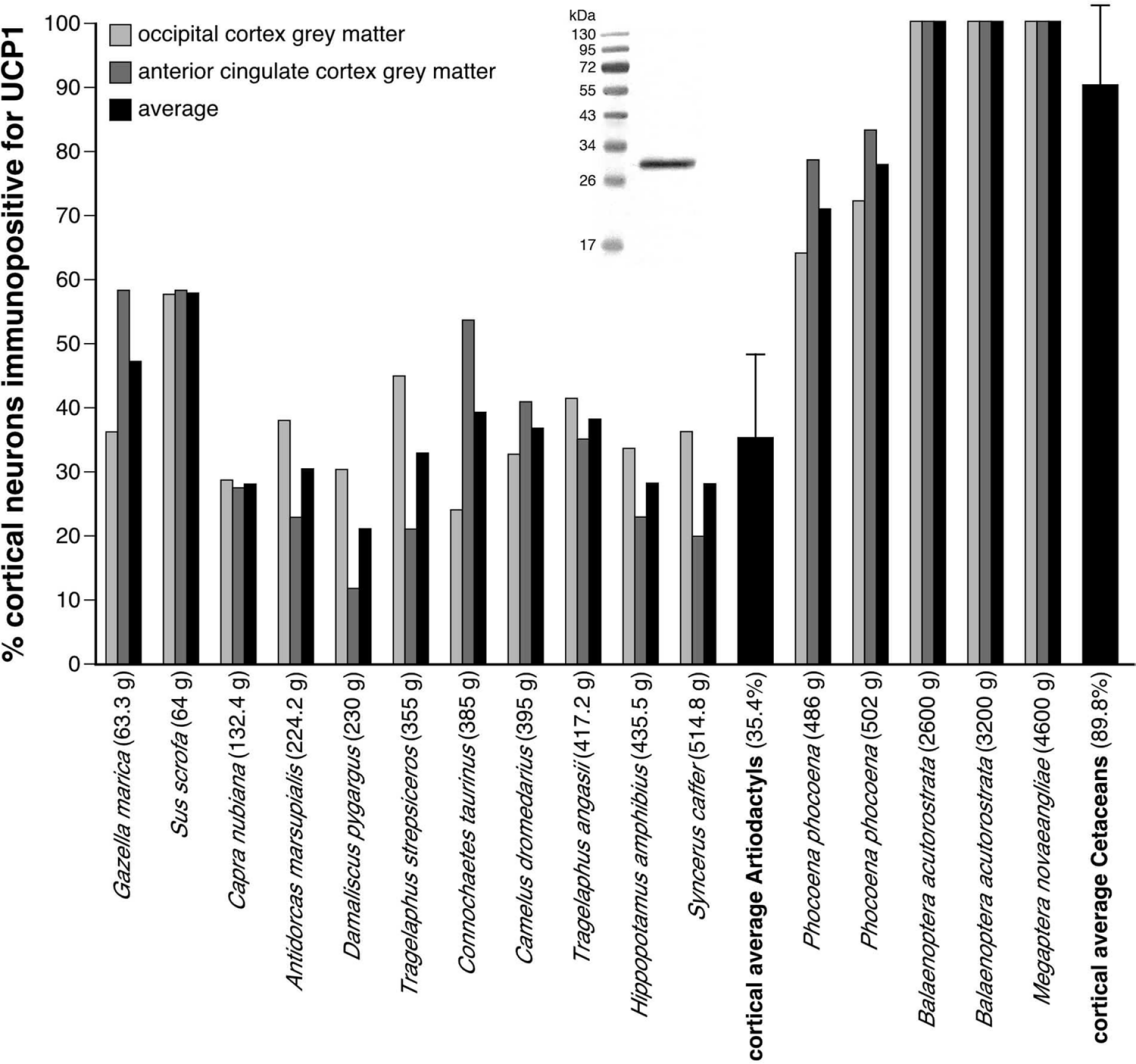
Quantification of UCP1 immunostaining in cetartiodactyl cerebral cortex. Graphical representation of the results of the stereological analysis of the percentage of cortical neurons immunopositive for UCP1 in the occipital and anterior cingulate cortices of the species studied. For each species the brain mass is given in grams next to the name on the x-axis. Note that the average percentage of cortical neurons immunopositive for UCP1 in the artiodactyls studied was 35.4%, while in the cetaceans studied it was 89.9% (Table 1, error bars on average bars represent one standard deviation). The Western immunoblot in the middle of the graph shows the specificity of the UCP1 antibody to brown fat taken from a laboratory rat (see Figure S6 for full-length unedited Western immunoblot).

**Figure 3:**
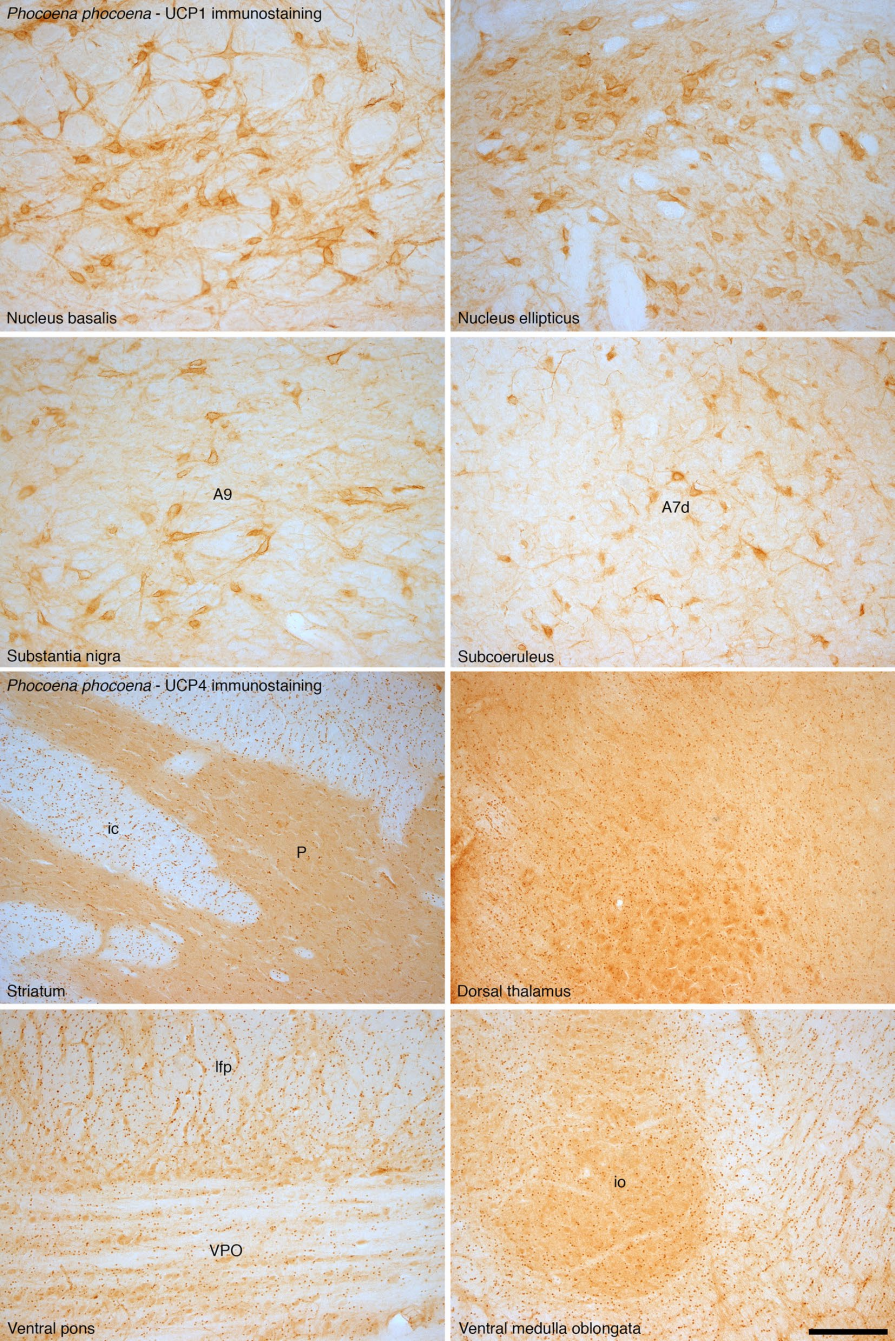
UCP1 and UCP4 immunostaining in non-cortical regions of the harbor porpoise brain. In addition to examining the expression of UCP1 and UCP4 in the cerebral cortex of the brain of the harbor porpoise, we examined several other brain regions. In all regions we found neurons with distinct UCP1 immunoreactivity, with an intracellular staining pattern similar to that observed in the neurons of the cerebral cortex. The photomicrographs shown here depict UCP1 immunostaining in various non-cortical regions of the harbour porpoise brain, including the nucleus basalis, nucleus ellipticus, the substantia nigra (A9), and the nucleus subcoeruleus (A7d, its diffuse region). In addition, in all regions we found glial cells with distinct immunoreactivity to the UCP4 antibody. Interestingly, the density of glial cells immunopositive for UCP4 appears higher in the white matter than in the grey matter, reflecting the same proportional distribution of stained glia as when comparing the white and grey matter of the cerebral cortex. The photomicrographs shown here depict UCP4 immunostaining in various non-cortical regions of the harbour porpoise brain, including the striatum (P—putamen, ic—internal capsule), dorsal thalamus, ventral pons (VPO—ventral pontine nucleus, lfp—longitudinal fasciculus of pons) and the ventral medulla oblongata (io—portion of inferior olivary nuclear complex). Scale bar = 250 µm and applies to all.

Employing immunohistochemical techniques, UCP1 immunolocalization was observed in neocortical neurons from the occipital and anterior cingulate cortical regions investigated in all cetartiodactyl species examined (Fig. 1, Table 1). Specificity of the UCP1 antibody was confirmed with Western blotting to brown fat taken from a laboratory rat (Fig. 2). UCP1 immunolabelling within the cortical neurons was observed in the perikaryal cytoplasm, as well as within the cytoplasm of the proximal portions of larger dendrites. The majority of the neurons immunopositive for UCP1 were pyramidal, although other cell types were also labelled (Fig. 1). Within the artiodactyls studied, neurons immunopositive for UCP1 were observed mainly in the subgranular layers of the cerebral cortex (IV, V and VI) with occasional labelled neurons being observed in the supragranular cortical layers (I, II and III). In contrast, UCP1-immunopositive neurons were observed throughout all layers of the cerebral cortex of the cetaceans studied. A systematic-random sampling analysis of the neurons immunopositive for UCP1 (Fig. 2, Table 1) revealed that the average percentage of neocortical neurons immunopositive for UCP1 in the artiodactyls studied was 35.4% (range: 11.86% in blesbok anterior cingulate cortex to 58.25% in domestic pig anterior cingulate cortex, Table 1). In contrast, an average of 89.8% of cortical neurons were immunopositive for UCP1 in the cerebral cortex of the cetaceans studied. The harbour porpoise (*Phocoena phocoena*) showed an average of 74.55% (range 71.28–83.19%) of cortical neurons being immunopositive for UCP1, while 100% of cortical neurons in the minke whale (*Balaenoptera acutorostrata*) and humpback whale (*Megaptera novaeangliae*) were immunopositive for UCP1 (Table 1). Using a two-proportions Z-test (as implemented in the R programming language) we tested the probability that the percentage of cortical neurons immunolabelled with UCP1 were equal in the artiodactyl and cetacean groups. Our analysis revealed that the proportion of immunolabelled UCP1 cortical neurons were significantly different between groups, with the cetaceans studied having a significantly higher proportion of UCP1-immunoreactive neurons in both the occipital cortex (χ^2^ = 56.30; *P* = 6.21 × 10^−14^) and anterior cingulate cortex (χ^2^ = 51.69; *P* = 6.49 × 10^−13^) than the artiodactyls studied. These observations imply that there has been a proportional increase of UCP1 expression in the cortical neurons of the cetaceans examined, to include almost all or all neurons of all layers, compared to the artiodactyls studied where UCP1 expression is limited to a smaller proportion of neurons mostly within the subgranular cortical layers. In addition, UCP1-immunostained neurons were found throughout all grey matter regions of the harbour porpoise brains examined (Fig. 3).

**Table 1:** Specimens used and data generated in the current study.

Species	Brain mass (g)	Neuronal density (mm^3^)		Grey matter glia density (mm^3^)		Glia:neuron ratio		White matter glia density (mm^3^)		% Grey matter neurons UCP1 immunopositive			
		AC	OC	AC	OC	AC	OC	AC	OC	AC	OC		
**Artiodactyls**													
*Gazella marica*	63.3	13,967	22,184	112,892	220,405	6.820	6.030	–	–	58.19	36.28		
*Sus scrofa*	64.0	18,355	23,808	108,238	133,283	5.897	5.598	273,326	230,191	58.25	57.69		
*Capra nubiana*	132.4	12,992	13,104	81,151	87,317	6.246	6.663	–	–	27.72	29.00		
*Antidorcas marsupialis*	224.2	11,808	13,887	69,440	86,121	5.881	6.202	–	–	23.12	38.21		
*Damaliscus pygargus*	230.0	19,632	28,189	83,679	105,486	3.740	4.260	283,841	250,063	11.86	30.35		
*Tragelaphus strepsiceros*	355.0	13,771	14,849	52,950	71,238	4.790	3.840	205,760	180,886	21.16	45.10		
*Connochaetes taurinus*	385.0	22,108	22,957	70,145	76,245	3.173	3.321	221,160	176,321	53.84	24.27		
*Camelus dromedarius*	395.0	15,635	9930	99,084	66,354	6.337	6.682	–	–	40.91	32.64		
*Tragelaphus angasii*	417.2	9432	10,718	65,040	87,719	6.896	8.184	–	–	35.13	41.60		
*Hippopotamus amphibius*	435.5	7519	8135	58,984	75,775	7.848	9.315	–	–	23.26	33.71		
*Syncerus caffer*	514.8	13,889	16,739	106,311	111,595	7.654	6.667	–	–	20.61	36.28		
**Cetaceans**													
*Phocoena phocoena*	486.0	10,938	20,129	97,623	153,415	7.620	8.930	210,627	187,064	78.59	63.96		
*Phocoena phocoena*	502.0	10,938	20,129	97,623	153,415	8.530	7.980	202,072	239,250	83.19	72.45		
*Balaenoptera acutorostrata*	2600.0	8671	11,119	82,149	114,384	9.470	10.290	160,172	175,982	100.00	100.00		
*Balaenoptera acutorostrata*	3200.0	8671	11,119	82,149	114,384	12.160	8.280	182,841	231,483	100.00	100.00		
*Megaptera novaeangliae*	4600.0	–	6922	–	183,231	–	10.220	–	183,231	100.00	100.00		

Species	Brain mass (g)	% Grey matter glia UCP4 immunopositive		% White matter glia UCP4 immunoreactive		Grey matter DBH bouton density (mm^3^)		White matter DBH bouton density (mm^3^)		Grey matter TH bouton density (mm^3^)		White matter TH bouton density (mm^3^)	
		AC	OC	AC	OC	AC	OC	AC	OC	AC	OC	AC	OC
**Artiodactyls**													
*Gazella marica*	63.3	0	0	0	0	8218	11,077	1325	1900	6132	9405	2538	1750
*Sus scrofa*	64.0	0	0	0	0	9257	10,682	2900	2988	5618	8595	1675	1013
*Capra nubiana*	132.4	0	0	0	0	6658	7229	2200	3025	6560	7561	1200	913
*Antidorcas marsupialis*	224.2	0	0	0	0	7699	7000	2350	2300	6347	7717	1675	1025
*Damaliscus pygargus*	230.0	0	0	0	0	7258	7856	2288	1288	4073	8135	1450	1450
*Tragelaphus strepsiceros*	355.0	0	0	0	0	6595	10,965	1688	2683	5077	10,097	2650	838
*Connochaetes taurinus*	385.0	0	0	0	0	6670	11,390	2188	2763	4763	9313	1363	675
*Camelus dromedarius*	395.0	0	0	0	0	6478	10,887	2675	3600	5785	10,815	2000	538
*Tragelaphus angasii*	417.2	0	0	0	0	10,278	11,233	2875	3788	7677	10,770	1950	1425
*Hippopotamus amphibius*	435.5	0	0	0	0	9867	12,305	3400	2488	8320	11,704	3288	2225
*Syncerus caffer*	514.8	0	0	0	0	8970	12,900	2513	2113	8172	8483	1400	863
**Cetaceans**													
*Phocoena phocoena*	486.0	33.27	31.84	63.45	47.17	11,763	16,013	2475	1613	13,973	18,247	1875	2200
*Phocoena phocoena*	502.0	32.74	33.58	69.17	45.06	11,567	15,898	2825	2063	14,092	17,802	1625	2088
*Balaenoptera acutorostrata*	2600.0	58.88	39.77	50.72	64.73	9156	13,488	4163	2138	11,786	15,010	1613	1738
*Balaenoptera acutorostrata*	3200.0	35.24	31.88	50.31	54.45	10,208	13,303	1513	4963	10,717	14,846	1875	1750
*Megaptera novaeangliae*	4600.0	–	29.33	–	58.97	–	–	–	–	–	–	–	–

Brain masses, neuronal densities, grey and white matter glia densities, grey matter glia:neuron ratio, percentage (%) of grey matter/white matter neurons/glia immunopositive to uncoupling proteins 1 and 4 (UCP1, UCP4), density of boutons immunoreactive for dopamine-B-hydroxylase (DBH) and tyrosine hydroxylase (TH) in the grey matter and white matter, in anterior cingulate cortex (AC) and occipital cortex (OC).

### UCP4/5 expression in cetacean glia

**Figure 4:**
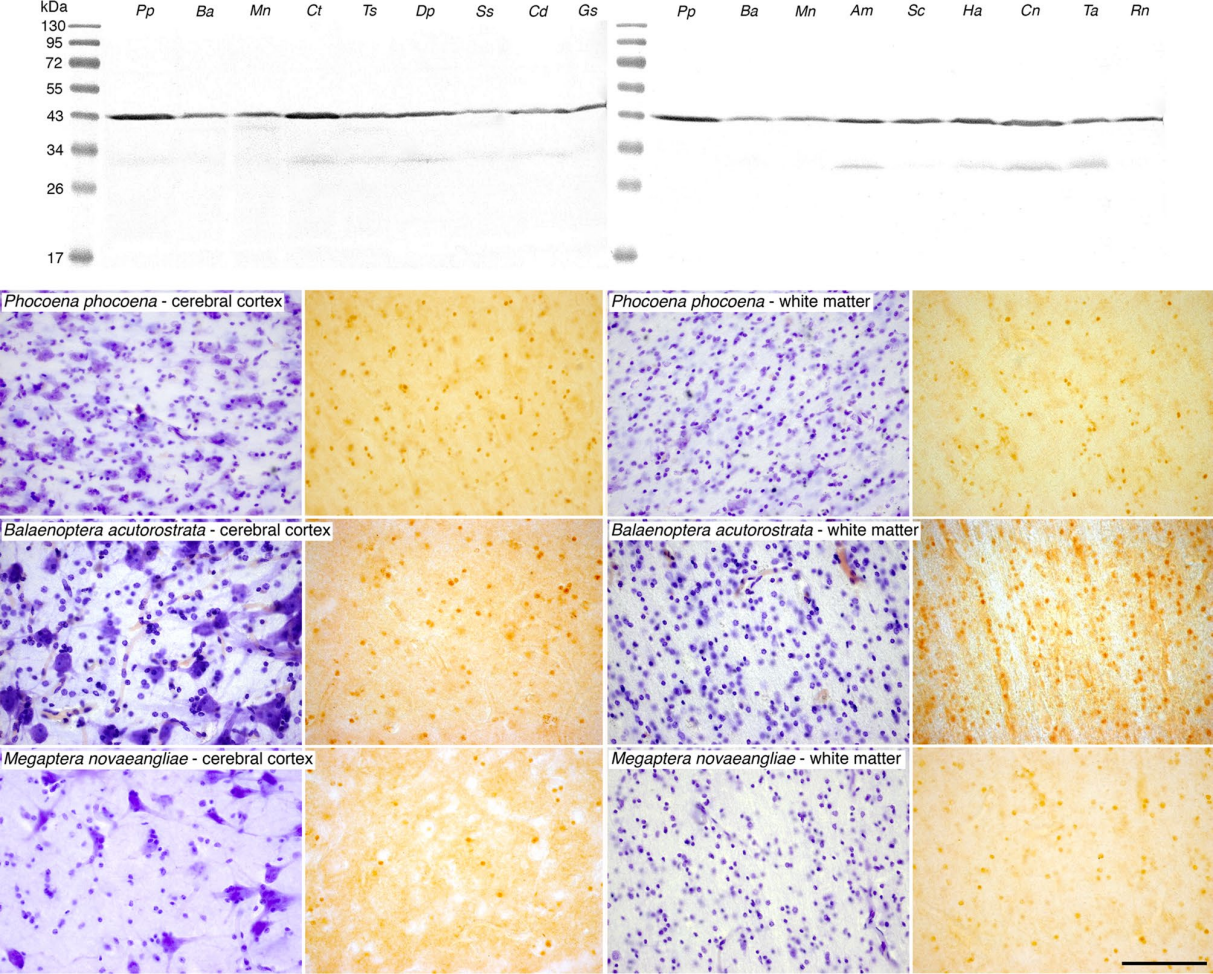
UCP4 Western blotting and immunostaining in cetartiodactyl cerebral cortex. While UCP4 was present in the cortical grey and white matter of all species, as evidenced in the Western blot at the top of the panel from the occipital cortex of all species studied, it was only found to be immunolocalized to glial cells in the cetaceans (See Figure S6 for full-length unedited Western immunoblots). Photomicrographs of Nissl-stained (purple colored images) and UCP4-immunostained (brown colored images) from cortical and subcortical white matter sections in a range of cetacean species. Note the presence of UCP4-immunoreactivity in approximately 30% of glial cells in the cerebral cortex and approximately 60% of glial cells in the white matter in all cetacean species (Table 1). The scale bar in the UCP4-immunostained section of *Megaptera novaeangliae*—white matter, equals 100 µm and applies to all photomicrographs. *Pp*—harbor porpoise, *Phocoena phocoena*; *Ba*—minke whale, *Balaenoptera acutorostrata*; *Mn*—humpback whale, *Megaptera novaeangliae*; *Ct*—blue wildebeest, *Connochaetes taurinus*; *Ts*—greater kudu, *Tragelaphus strepsiceros*; *Dp*—blesbok, *Damaliscus pygargus*; *Ss*—domestic pig, *Sus scrofa*; *Cd*—dromedary camel, *Camelus dromedarius*; *Gm*—sand gazelle, *Gazella marica*; *Am*—springbok, *Antidorcas marsupialis*; *Sc*—African buffalo, *Syncerus caffer*; *Ha*—river hippopotamus, *Hippopotamus amphibius*; *Cn*—Nubian ibex, *Capra nubiana*; *Ta*—nyala, *Tragelaphus angasii*; *Rn*—laboratory rat, *Rattus norvegicus*.

UCP4 has been identified using Northern (RNA) blots in the human brain and is suggested to play a role in thermogenesis^19^. Using Western blots on tissue from the occipital and anterior cingulate cortical regions investigated, we found evidence for the presence of UCP4 in the brains of all artiodactyl and cetacean species studied (Fig. 4). In contrast to the detectable presence of UCP4 with Western blotting, immunohistochemical localization of UCP4 was only observed in the cetacean brains. In all three cetacean species studied, we observed strong immunolocalization of UCP4, and weaker immunolocalization of UCP5, within glial cells in the cerebral cortex and the subcortical white matter from the occipital and anterior cingulate cortical regions investigated, but no staining of neurons (Fig. 4, Table 1). In the harbour porpoise an average of 33.16% of glial cells in the cerebral cortical grey matter (from anterior cingulate and occipital regions) were immunopositive for UCP4, while an average of 57.12% of glial cells in the cortical white matter (from anterior cingulate and occipital regions) were immunopositive for UCP4. In the minke whale an average of 41.44% of glial cells in the cortical grey matter and an average of 55.05% of glial cells in the cortical white matter were immunopositive for UCP4. In the humpback whale an average of 29.33% of glial cells in the cortical grey matter and an average of 58.97% of glial cells in the cortical white matter were immunopositive for UCP4. Thus, in the cetaceans studied, approximately 36% of glial cells in the cortical grey matter and 56% of glial cells in the cortical white matter show specific immunolocalization of UCP4 (Table 1). In all three cetacean species UCP5 was also expressed in similar proportions of glial cells, but the strength of immunostaining was substantially weaker. A limited examination of the immunolocalization of UCP4 and UCP5 in other regions of the harbour porpoise brain showed similar levels of glial staining in both grey and white matter (Fig. 3), indicating that UCP4 and UCP5 are proteins likely to be expressed in glial cells throughout the harbour porpoise brain. Based on these observations, we conclude that while UCP4 and UCP5 are proteins found in the brains of both the artiodactyl and cetacean species studied, in these cetaceans they exhibit a specific localization to glial cells, indicating a specialization in their expression, and related function.

### Noradrenergic bouton density in cetacean cerebral cortex

**Figure 5:**
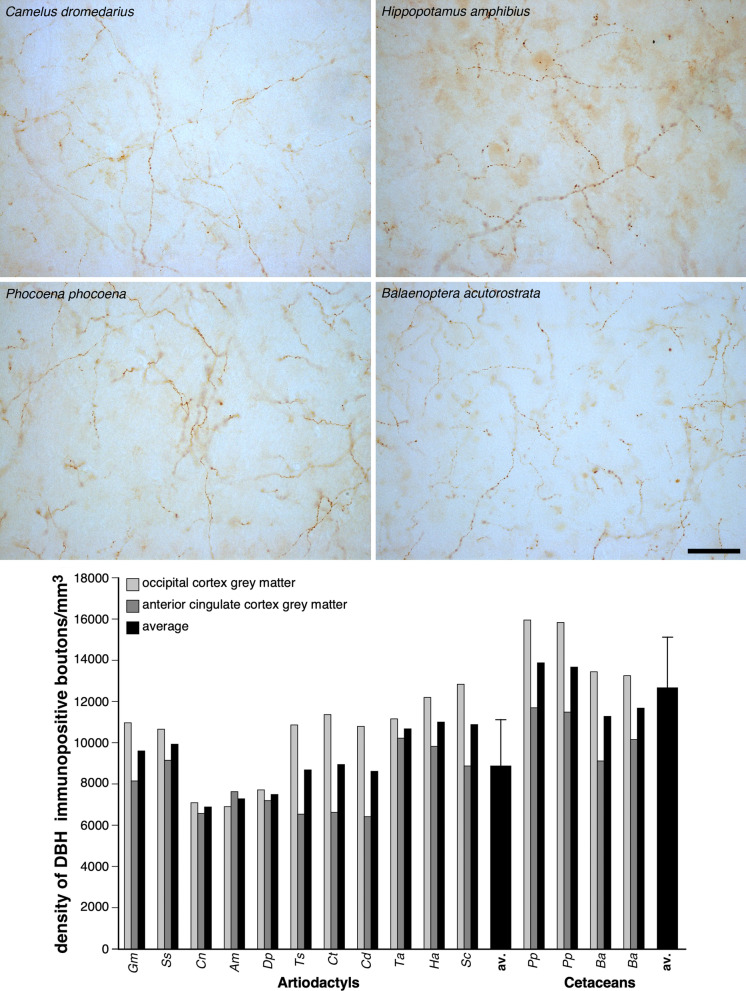
Quantification of noradrenergic bouton density in cetartiodactyl cerebral cortex. Photomicrographs of dopamine-ß-hydroxylase (DBH)-immunostained axonal boutons in the occipital cortical grey matter of *Camelus dromedarius*, *Hippopotamus amphibius*, and *Phocoena phocoena*, and the anterior cingulate cortical grey matter of *Balaenoptera acutorostrata*. The scale bar = 50 µm and applies to all photomicrographs. Note the higher density of the DBH-immunoreactive boutons in the cortical grey matter of cetaceans compared to the artiodactyls as confirmed with stereological analysis (see the graph below the photomicrographs), showing that the density of DBH-immunoreactive boutons in the cortical grey matter of cetaceans is, on average, 1.4 times higher than that observed in artiodactyls (Table 1, error bars on average bars represent one standard deviation). *Gm*—sand gazelle, *Gazella marica*; *Ss*—domestic pig, *Sus scrofa*; *Cn*—Nubian ibex, *Capra nubiana*; *Am*—springbok, *Antidorcas marsupialis*; *Dp*—blesbok, *Damaliscus pygargus*; *Ts*—greater kudu, *Tragelaphus strepsiceros*; *Ct*—blue wildebeest, *Connochaetes taurinus*; *Cd*—dromedary camel, *Camelus dromedarius*; *Ta*—nyala, *Tragelaphus angasii*; *Ha*—river hippopotamus, *Hippopotamus amphibius*; *Sc*—African buffalo, *Syncerus caffer*; av. —average; *Pp*—harbor porpoise, *Phocoena phocoena*; *Ba*—minke whale, *Balaenoptera acutorostrata.*

As one of the many known functions of noradrenaline (NA) is to control concentrations of UCPs and initiate UCP activity in brown adipocytes^24,25^, we used immunohistochemical staining for dopamine-β-hydroxylase (DBH, the enzyme that converts dopamine to noradrenaline in the catecholamine biosynthetic pathway) to examine the density of noradrenergic boutons in the grey and white matter of the cerebral cortex (from anterior cingulate and occipital regions) in the cetartiodactyl species studied (Fig. 5; Table 1). The average density of NA boutons in the cortical grey matter of the artiodactyls studied was 8980 boutons/mm^3^ (range: 6478/mm^3^ in dromedary camel anterior cingulate cortex to 12,900/mm^3^ in African buffalo occipital cortex). In the cortical grey matter of the cetaceans studied, an average density of 12675 NA boutons/mm^3^ was observed (range: 9156/mm^3^ in minke whale anterior cingulate cortex to 16013/mm^3^ in harbour porpoise occipital cortex, Table 1). Using a two sample T-test we compared DBH-immunoreactive bouton density in the grey matter of the anterior cingulate and occipital cortex between artiodactyls and cetaceans studied. The cetaceans studied have significantly higher DBH-immunoreactive bouton densities in both the anterior cingulate and occipital cortex compared to the artiodactyls studied (anterior cingulate: *t* = − 3.595; df = 15, *P* = 0.011; occipital cortex: *t* = − 4.546; df = 15, *P* = 0.002). In the cortical white matter, an average density of 2515 NA boutons/mm^3^ was observed in the artiodactyls studied, which was not significantly different to (anterior cingulate: *t* = − 0.5977; df =15, *P* = 0.585; occipital: *t* = − 0.08; df =15, *P* = 0.941) the average NA bouton density found in the cortical white matter of the cetaceans studied (2719 NA boutons/mm^3^, Table 1, Supplementary Figure S1). When a third variable, such as cortical neuron density, cortical glia density or brain mass (Table 1) were analysed with the current data using analysis of covariance (ANCOVA), the cetaceans studied were still observed to have statistically significantly higher DBH-immunoreactive bouton densities in the cortical grey matter than the artiodactyls studied. Thus, in addition to having an amplified (UCP1) and localized (UCP4/5) representation of UCPs in the cortical grey matter, the cetaceans studied have a significantly denser noradrenergic innervation, which likely functions to increase concentrations of, and activate, UCPs. Quantitative analysis of bouton densities following immunohistochemical staining for tyrosine hydroxylase (TH, the enzyme that converts tyrosine to L-3,4-dihydroxyphenylalanine in the catecholamine biosynthetic pathway) provided similar results (Supplementary Figures S2, S3, S4).

## Discussion

Our observations indicate, based on the data derived from the individual cetacean brains studied herein, and which is likely to be characteristic of all cetacean brains, that the cetacean brain appears to have three augmented characteristics of a pre-existing, brain-based, non-shivering thermogenic system that should increase heat generation capabilities above and beyond that seen in the brains of the closely related artiodactyls. First, the expanded expression of UCP1 throughout almost all cortical neurons indicates that, unlike in artiodactyls, the majority of cortical neurons within the cetacean brain can function as thermogenic units if necessary. Second, the localization of UCP4/5, within many glial cells of the cetacean brain, indicates that between 30 and 70% of glial cells may be employed as thermogenic units in both grey and white matter if necessary. The generally higher density of glial cells and the higher glia:neuron ratio in the cetacean brain (Table 1) indicates that glial based UCPs may form a potentially powerful thermogenic mechanism in the cetacean brain. Last, the increased density of noradrenergic boutons in the cetacean cerebral cortex compared to the artiodactyls indicates that the capacity to increase concentrations of UCP within the tissue and activate these proteins appears to be enhanced in the cetaceans compared to the artiodactyls. As cetaceans undergo unihemispheric slow wave sleep (USWS) without rapid eye movement sleep^12^, and have a very higher number of noradrenergic neurons in the locus coeruleus complex^27,28^ (Supplementary Figure S5), the cetacean brain is likely to have a constant supply of noradrenaline, which will not occur in the artiodactyls, again enhancing the potential for thermogenesis by the cetacean brain. This link between noradrenaline and thermogenesis in the cetacean brain is supported by the observation that during cetacean USWS, when the activity of the noradrenergic neurons of the locus coeruleus complex (Supplementary Figure S5) is reduced unilaterally^29^, the temperature of the ipsilateral sleeping hemisphere gradually decreases^12^. In summary, neuronal and glial portions of the cetacean brain appear to have specializations associated with UCPs, and the increased noradrenergic input should act to increase the concentration of these proteins in the tissue and activate them, indicating that amplified thermogenetic capabilities are likely to be an extremely important basic function of the cetacean central nervous system^6^.

The findings presented herein support the thermogenesis hypothesis of cetacean brain evolution and function^6,7^. The presence of UCPs in the majority of cortical neurons as well as within a substantial proportion of glial cells, together with the associated increased of noradrenergic innervation throughout the grey and white matter of the brain is important, because in situations of thermal challenge, which in the case of cetaceans would be continuous^6^, the neurons and glia could be recruited to drive thermogenic processes, in addition to the functions normally associated with these cell types. This proposal is consistent with the known anatomical variances of the cetacean brain compared with other mammals^4,6,8–10^, the physiology and anatomy of cetacean sleep^12,13,27,28^, and the pragmatic view of cetacean behaviour^5,7,14^. Thus, we conclude that while the cetacean brain obviously provides adequate neural/cognitive processing to sustain life, it also exhibits the biological features that would allow it to produce sufficient heat to prevent suboptimal performance of the brain while under constant thermal challenge.

The brains of cetaceans became both relatively and absolutely large around 20 million years after the ancestors of the modern cetacean fauna, the Archaeocetes, were already obligatory aquatic species. This enlargement in brain size occurred at the Archaeocete to Neocete (modern cetacean fauna) faunal transition approximately 32 million years ago (mya)^6,7^. Since this transition, the relative size of the brains of the Neocete have, for the most part, remained consistent^6^, although studies indicate a secondary increase in the relative size of the brain of delphinid cetaceans^30^. The thermogenetic hypothesis of cetacean brain evolution posits that, as this specific time point in the evolution of cetaceans (32 mya) coincides with significant drops in oceanic water temperatures as well as the loss of the warm, shallow, nutrient-rich Tethys sea^31–33^, the enlargement of the cetacean brain is an adaptive response to environmental thermal challenges^6,7^. Thus, water temperature can be posited to be the ultimate evolutionary pressure initiating the increase in brain size in cetaceans. This increase in brain size can be hypothesized to be the outcome of the combinatorial action of three specific factors^6^: (1) the rate of heat loss to water makes it imperative that neonatal cetaceans have a birth mass greater than 6 kg or risk death due to hypothermia^34^ (notably, neonatal cetaceans do not appear to sleep for their first three months of life^13^, the muscular activity generating heat to offset the risk of hypothermia); (2) an allometric scaling law of form in eutherian mammals shows that neonatal body mass is primarily determined by maternal body mass^6^, meaning that the adult cetaceans will need to be large bodied in order to produce neonates with body masses greater than 6 kg, and generally, across mammalian species, larger bodied mammals tend to have larger brains^6^; and (3) in extant cetaceans, the relative size of the brain is strongly correlated with the range of water temperatures they inhabit^6^. As the mammalian brain produces its own heat^16–18^, and cetacean brains are under constant thermal pressure, the findings of the current study indicate that the concurrent evolution of a specialized neurothermogenic system in the cetacean brains evolving a larger size, is likely to be a crucial, or even a prerequisite, trait for cetaceans to overcome the ubiquitous thermal environmental pressures they experience. Specializations of brown adipose tissue in cetacean blubber associated with the expression of uncoupling proteins, specifically UCP1^35^, indicates that both the body and the brain of extant cetaceans have evolved enhanced thermogenetic mechanisms through specializations of pre-existing non-shivering thermogenetic mechanisms that form part of the basic physiology of endothermic mammals.

Genetic studies on the cetacean nervous system demonstrate three major points of importance to our understanding of cetacean brain evolution^36^. First, 27 genes associated with the nervous system appear to have been positively selected for in the cetacean lineage including those specifically involved in sleep^36^, coinciding with the unusual sleep physiology of cetaceans that appears, in part, to be related to thermogenesis^12,13^. Second, there appears to have been an ebb in the accumulation of genetic changes associated with the nervous system in the cetacean lineage^36^, which coincides with the stasis of the relative and absolute brain size of cetaceans following the Archaeocete–Neocete faunal transition^6,7^. Third, seven mitochondrial expressed genes underwent positive selection in the cetacean lineage^36^, which coincides with the amplification and specialization of the expression of the UCP proteins 1, 4 and 5 shown herein. However, the role of UCPs in non-shivering thermogenesis within the mammalian brain is not unequivocally established^37^. It has been reported that *ucp1* and the associated enhancer is inactivated/lost/pseudogenized in some, but not all, cetacean species^38–40^, although these studies have not examined the physiology and functionality of the UCP1 protein in cetaceans, and there are contradictory results between studies in terms of which cetacean species have modifications of *ucp1*. The *ucp1* variances reported appear to be primarily frameshifts at the start of the codon, along with other minor modifications, which may not necessarily result in either inactivation or functional alteration of the proteins produced^41^. Given the presence of the UCP1 protein in cetacean brains as shown with immunohistochemistry and in the brown adipose tissue of cetaceans, including delphinid species, using both immunohistochemistry and Western blot analysis^35^, it is possible that the variations in *ucp1* observed with data-mining techniques represent specializations, rather than pseudogenization, which would be in agreement with the UCP protein specializations observed in both the brain and brown adipose tissue^35^ of the cetaceans studied and support the proposal that the UCP proteins in cetaceans are involved in the process of non-shivering thermogenesis in both the brain and body.

Thus, palaeoneurological, palaeoclimatological, genomic, neuroanatomical, neurochemical and neurophysiological studies of cetaceans all converge upon the concept that thermal pressures during the Archaeocete–Neocete faunal transition underlie the historical enlargement and current functionalities of the cetacean brain^6,7^. This understanding of the evolution and functionality of the cetacean brain, which is reflected in their biogeographical distribution^6^, may be of importance in providing a level of predictability to potential changes in the zoogeography of extant cetaceans in the face of rising ocean heat content associated with climate change^42^.

The present study has broad reaching implications in terms of our understanding of the evolution of large brain size in mammals, including humans. By illustrating that it is possible to evolve a large brain for reasons not necessarily associated with a need for greater cognitive complexity, indicates that we should reassess our narratives regarding the evolution of large brains in humans, elephants and other mammals. In all of these situations, alternative explanations for increased brain size can be posited^43,44^. Most importantly, the current study emphasizes that, in terms of brain evolution and the resultant outcome, the starting point, this being what the brains of the ancestral species were like prior to enlargement, and any major environmental changes that occurred, are likely to be the best predictor of the functionality of the brain after enlargement. For extant cetaceans, the starting point was the archaeocete brain, which, for animals that could grow to over 14 m in length, had a diminutive cerebral cortex with a surface area of around 50 cm^2^ in total^6^. On the other hand, the human brain evolved from an Australopithecine starting point, with brains quite similar to those seen in modern great apes, and thus the comparatively remarkable cognitive capacities of modern humans can be attributed in part to the enlargement of this ancestral brain, with the associated increases in neuronal complexity^43^.

## Materials and methods

### Specimens

We used brains obtained from three cetacean species (harbour porpoise—*Phocoena phocoena*, minke whale—*Balaenoptera acutorostrata*, and humpback whale—*Megaptera novaeangliae*) and 11 artiodactyl species (sand gazelle—*Gazella marica*, domestic pig—*Sus scrofa*, Nubian ibex—*Capra nubiana*, springbok—*Antidorcas marsupialis*, blesbok—*Damaliscus pygargus*, greater kudu—*Tragelaphus strepsiceros*, blue wildebeest—*Connochaetes taurinus*, dromedary camel—*Camelus dromedarius*, nyala—*Tragelaphus angasii*, river hippopotamus—*Hippopotamus amphibius*, and African buffalo—*Syncerus caffer*) (Table 1). All artiodactyl brains were perfusion fixed with 4% paraformaldehyde in 0.1 M phosphate buffer through the carotid arteries following euthanasia^45^. The harbour porpoise specimens were perfusion fixed through the heart following euthanasia, while the minke whale and humpback whale brains were immersion fixed in 4% paraformaldehyde in 0.1 M phosphate buffer. All brains were then stored in an antifreeze solution at – 20 °C until use^45^. All specimens were taken under appropriate governmental permissions, with ethical clearance provided by the University of the Witwatersrand Animal Ethics Committee (Clearance number 2008/36/1), which uses guidelines similar to those of the National Institutes of Health regarding the use of animals in scientific research and is compliant with ARRIVE guidelines.

### Immunohistochemical staining

Blocks of tissue from the anterior cingulate (dorsal to the rostrum of the corpus callosum, in all species apart from the humpback whale where we did not have this tissue block) and occipital cortex (presumably primary visual cortex, from all species) with underlying white matter were taken from each of the specimens. These were placed in a 30% sucrose in 0.1 M phosphate buffer solution at 4 °C until equilibrated. The blocks were frozen in crushed dry ice, mounted on an aluminium stage and sectioned at 50 µm orthogonal to the pial surface. Alternate sections were stained for Nissl (with 1% cresyl violet), UCP1, UCP2, UCP3, UCP4, UCP5, dopamine-ß-hydroxylase (DBH) and tyrosine hydroxylase (TH). To investigate the presence of neural structures immunolocalizing uncoupling proteins, DBH and TH, we used standard immunohistochemical procedures with antibodies directed against UCP1, UCP2, UCP3, UCP4, UCP5, DBH and TH. While immunolocalization for UCP1, UCP4, UCP5, DBH and TH were clear, only occasional cortical neurons were immunopositive for UCP2, and no immunolocalization could be detected for UCP3 in the species studied. It should be noted here that immunostaining for DBH and TH did not work in the humpback whale specimen, perhaps due to the fixation procedure or the conformation of the targeted proteins in this species preventing recognition of the DBH and TH proteins by the antibodies used. Sections used for the Nissl series were mounted on 0.5% gelatine-coated glass slides, cleared in a solution of 1:1 chloroform and absolute alcohol, then stained with 1% cresyl violet to reveal cell bodies. For the immunohistochemical staining, each section was treated with endogenous peroxidase inhibitor (49.2% methanol:49.2% 0.1 M PB:1.6% of 30% H_2_O_2_) for 30 min and subsequently subjected to three 10 min 0.1 M PB rinses. Sections were then incubated for 2 h, at room temperature, in blocking buffer (containing 3% normal rabbit serum, NRS, for the UCP1-5 sections/3% normal horse serum, NHS, for the DBH sections/3% normal goat serum, NGS, for the TH sections, plus 2% bovine serum albumin and 0.25% Triton-X in 0.1 M PB). This was followed by three 10 min rinses in 0.1 M PB. The sections were then placed in the primary antibody solution that contained the appropriately diluted primary antibody in blocking buffer for 48 h at 4°C under gentle shacking. The optimal dilutions for the UCP primary antibodies were determined with a series of stains in which the dilution of the primary antibodies ranged from 1:300 through to 1:9600, with any staining in all species being absent at a dilution of 1:4800 irrespective of fixation method. We used antibodies directed against UCP1 (Santa Cruz Biotechnology, C-17, sc-6528, Lot# D0411, goat polyclonal IgG, dilution 1:300, RRID:AB_2304265), UCP2 (Santa Cruz Biotechnology, C-20, sc-6525, Lot# E0211, goat polyclonal IgG, dilution 1:300, RRID:AB_2213585), UCP3 (Santa Cruz Biotechnology, C-20, sc-7756, Lot# A2511, goat polyclonal IgG, dilution 1:300, RRID:AB_2213922), UCP4 (Santa Cruz Biotechnology, N-16, sc-17582, Lot# E2004, goat polyclonal IgG, dilution 1:300, RRID:AB_793648), UCP5 (Santa Cruz Biotechnology, Q-16, sc-50540, Lot# B1207, goat polyclonal IgG, dilution 1:300, RRID:AB_2286101), DBH (Merck-Millipore, MAB308, mouse monoclonal IgG, dilution 1:4000, RRID:AB_2245740) and TH (Merck-Millipore, AB151, rabbit polyclonal IgG, dilution 1:3000, RRID:AB_10000323). This incubation was followed by three 10 min rinses in 0.1 M PB and the sections were then incubated in a secondary antibody solution (1:1000 dilution of biotinylated anti-goat IgG, BA-5000, Vector Labs, for UCP1-5 sections/1:1000 dilution of biotinylated anti-mouse IgG, BA 2001, Vector labs, for DBH sections/1:1000 dilution of biotinylated anti-rabbit IgG, BA-1000, Vector Labs, for TH sections, in a blocking buffer containing 3% NRS/NHS/NGS and 2% BSA in 0.1 M PB) for 2 h at room temperature. This was followed by three 10 min rinses in 0.1 M PB, after which sections were incubated for 1 h in avidin-biotin solution (at a dilution of 1:125, Vector Labs), followed by three 10 min rinses in 0.1 M PB. Sections were then placed in a solution of 0.05% 3,3′-diaminobenzidine (DAB) in 0.1 M PB for 5 min, followed by the addition of 3 ml of 3% hydrogen peroxide to each 1 ml of solution in which each section was immersed. Chromatic precipitation was visually monitored and verified under a low power stereomicroscope. Staining was allowed to continue until such time as the background stain was at a level that would assist architectural reconstruction and matching without obscuring the immunopositive neurons. Development was halted by placing the sections in 0.1 M PB, followed by two more rinses in 0.1M PB. To test for non-specific staining of the immunohistochemical protocol, in selected sections the primary antibody or the secondary antibody were omitted, which resulted in no staining of the tissue. The immunostained sections were then mounted on 0.5% gelatine coated glass slides, dried overnight, dehydrated in a graded series of alcohols, cleared in xylene and coverslipped with Depex. Digital photomicrographs were captured using Zeiss Axioshop and Axiovision software. No pixilation adjustments, or manipulation of the captured images were undertaken, except for the adjustment of contrast, brightness, and levels using Adobe Photoshop 7.

### Western immunoblotting

Protein expression for UCP1 and UCP4 was assayed using standard qualitative Western immunoblotting techniques. To verify the specificity of the UCP1 antibody for the UCP1 protein, we tested this antibody with rat brown fat. For the UCP4 antibody protein samples were extracted from the paraformaldehyde fixed tissue using the Qproteome FFPE Tissue Kit (Qiagen, Germany). The tissue blocks analysed here were taken from the anterior cingulate and occipital cortex (as described above) and contained both gray and white matter. 30–40 mg of the sample were incubated in 100 µl of Extraction Buffer EXB Plus (Qiagen, Germany) containing 6% β-mercaptoethanol on ice for 5 min and mixed by vortexing. The samples were boiled for 20 min at 100°C and subsequently incubated at 50°C overnight with agitation at 300 rpm. The samples were then placed on ice for 1 min and centrifuged for 15 min at 14 000g at 4°C. The supernatant was transferred into clean tubes and the protein concentration was determined using the Bradford protein assay kit (Bio-Rad Laboratories, USA). The protein extracts (20 µg) were made soluble in sample buffer comprised of 0.0625 M Tris–HCl, pH 6.8, 10% glycerol, 2% SDS, 2.5% β-mercaptoethanol and 0.001% bromophenol blue, boiled at 95°C for 5 min and subjected to 12% SDS-polyacrylamide gel electrophoresis and transferred to polyvinylidene difluoride (PVDF) (Millipore) at 20 V/cm for 1h. Electrophoresis and protein transfer was achieved using Mini Trans-Blot Electrophoretic Transfer Cell (Bio-Rad Laboratories, Inc. USA). After the transfer the blots were blocked for 2 h in 1 × Animal-Free Blocker (SP-5030 Vector Labs, USA). The blots were incubated over night at 4°C under gentle agitation in the primary antibody solutions (1:300 goat anti-UCP1, Santa Cruz Biotechnology, sc-6528 or 1:300 goat anti-UCP4, Santa Cruz Biotechnology, sc-17582). The blots were washed for 3 × 10 min in 1 × Animal-Free Blocker and incubated for 1 h at room temperature in HRP-conjugated rabbit anti-goat secondary antibody (1:1000, Dako, USA) for 1 h. This was followed by 3 × 10 min washes with 50 mM Tris buffer, pH 7.2. The protein bands were detected using 3,3′-diaminobenzidine tetrahydrochloride hydrate (DAB) (Sigma, D5637). The blots were incubated in a solution containing 1mg/ml DAB in 50 mM Tris, pH 7.2 for 5 min at room temperature, followed by the addition of an equal amount of 0.02% hydrogen peroxide solution. Development was arrested by placing the blots in 50 mM Tris (pH 7.2) for 10 min, followed by two more 10 min rinses in distilled water.

### Stereological analysis

Using a design-based stereological approach we analysed immunohistochemically stained sections in the grey matter of the anterior cingulate and occipital cortex, as well as the underlying white matter from these regions of 14 cetartiodactyl species. Regions of interest (ROI) were drawn from similar locations across species as supported by published anatomical descriptions of the cetacean and artiodactyl brain. Using a light microscope equipped with a motorized stage, digital camera, MicroBrightfield system (MBF Bioscience, USA) system and StereoInvestigator software (MBF Bioscience, version 2018.1.1; 64-bit), we quantified UCP1-immunoreactive neuron densities in the grey matter, UCP4-immunoreactive glia densities in the grey and white matter, and DBH- and TH-immunoreactive bouton densities in the grey and white matter of these cortical regions. Separate pilot studies for each immunohistochemical stain was conducted to optimise sampling parameters, such as the counting frame and sampling grid sizes, and achieve a coefficient of error (CE) below 0.1^27,46–49^. In addition, we measured the tissue section thickness at every sampling site, and the vertical guard zone was determined according to tissue thickness to avoid errors/biases due to sectioning artefacts^27,46–49^. Supplementary Tables S1–S4 provide details of the parameters used for each neuroanatomical region and stain and between the species in the current study. To estimate the ROI total number, we used the ‘Optical Fractionator’ probe.

UCP1- and UCP4-immunoreactive neuron and glia densities were obtained by sampling the cortical areas of interest and subjacent white matter with the aid of an optical disector. The cortex and white matter were outlined separately at low magnification (2X), and the optical disector was performed at 40X. UCP-immunoreactive neuron and glia density was calculated as the total number of UCP-immunoreactive neurons and glia divided by the product of surface area (x, y), the tissue sampling fraction, and the sectioned thickness (50 µm). The tissue sampling fraction was calculated as the ratio of the optical disector height to mean measured section thickness. Given that overall cell density per unit volume is known to vary with differences in brain size, we calculated the percentage of UCP-immunoreactive neurons or glia, expressed as the ratio of UCP-immunoreactive neurons or glia to total neuronal or glial density for each region of interest, to standardize the data for cross species comparison. Using Nissl-stained sections we obtained estimates of neuronal and glial densities within the cortex and glial density within the white matter using optical disector probes combined with a fractionator sampling scheme^46^. A pilot study determined the optimal sampling parameters and grid dimensions to place disector frames in a systematic-random manner. For DBH and TH bouton densities, ‘spot’ densities were calculated by multiplying the ROI area by the cut section thickness, and then using the generated volume as the denominator to the ROI estimated number. For all tissue sampled the optical fractionator was used while maintaining strict criteria, e.g. only complete boutons were counted, 63 X oil immersion, and obeying all commonly known stereological rules. The stereologic analyses presented here resulted in sampling an average of 118 counting frames per region of interest with a total of 13,053 counting frames investigated.

### Statistical analyses

We hypothesized that the percentage of cortical neurons immunoreactive to UCP1 were significantly different between artiodactyls and cetaceans. To test this hypothesis, we compared the proportion of UCP1 expression in the anterior cingulate and occipital cortex of 16 cetartiodactyls. For the anterior cingulate cortex, we sampled a total of 1109 sampling sites (~ 100 sites per species) within the artiodactyl group and found that 36.83% of sampled cortical neurons were immunoreactive to UCP1. In comparison our cetacean sample consisted of 723 sampling sites (~ 145 sites per species), with 87.28% of the sampled cortical neurons immunoreactive to UCP1. For the occipital cortex, we sampled a total of 1 038 sites (~ 94 sites per species) within the artiodactyl group and found that 34% of sampled cortical neurons within the occipital cortex were immunoreactive to UCP1. The cetacean sample consisted of 723 sampling sites (~ 145 sites per species), and we found that 92.36% of the sampled cortical neurons were immunoreactive to UCP1.

To test if the respective underlying proportions were different between the sample groups, we conducted statistical hypothesis testing using the Two-Proportions Z-test as implemented in the R Programming language. Our Null hypothesis (*H*_o_) stated that there is no significant difference between the proportions of artiodactyl immunoreactive UCP1 sampled cortical neurons (π_1_) and the proportions of cetacean UCP1 sampled cortical neurons (π_2_)—that is, π_1_ − π_2_ = 0. The alternate hypothesis (*H*_1_) stated that there is a significant difference in these proportions such that π_1_ − π_2_ ≠ 0, with one of the proportions being either less than or greater than the other. We thus conducted a two-sided hypothesis test, with the significance level (α) set at 0.05 (i.e., *P*-values less than, or equal to, α, would reject the null hypothesis in favour of the alternate hypothesis). Based on these analyses the proportion of immunolabelled UCP1 cortical neurons were found to be significantly different between the groups, with cetaceans having a significantly higher proportion of UCP1-immunoreactive neurons in the anterior cingulate cortex (χ^2^ = 51.69; df =1, *P* = 6.49 × 10^−13^, 95% confidence interval = − 0.122; − 0.067) and occipital cortex (χ^2^ = 56.30; *P* = 6.21 × 10^−14^, 95% confidence interval = − 0.114; − 0.060).

We used a two sample T-test (as implemented in R) to test for significant differences in noradrenergic bouton density between cetaceans and artiodactyls. Cetaceans were found to have significantly higher mean DBH-immunoreactive bouton densities in the anterior cingulate cortex as compared to artiodactyls (*t* = − 3.595; df =15, *P* = 0.011). Cetaceans were also found to have significantly higher mean DBH-immunoreactive bouton densities in the occipital cortex as compared to artiodactyls (*t* = − 4.546; df =15, *P* = 0.002). Similarly, we tested for significant differences in mean DBH bouton density in the underlying cortical white matter of cetaceans and artiodactyls. We did not find any significant differences in DBH-immunoreactive bouton density for the anterior cingulate (*t* =− 0.597; df =15, *P* = 0.585) or occipital cortex (*t* = − 0.08; df =15, *P* = 0.941).

To test for the effect of confounding variables on the significant differences observed in DBH bouton density in the cortex, we used an analysis of covariance controlling sequentially for the effect of cortical neuron density, cortical glia density and brain mass. Our analyses revealed that after adjusting for the density of cortical neurons cetaceans still had significantly higher DBH-immunoreactive bouton density in the anterior cingulate cortex (adjusted mean = 10.176) in comparison to artiodactyls (adjusted mean = 8.176) (*F* = 5.222; df =13, *P* = 0.041). Adjusting for the covariate cortical neuron density, resulted in a similar result for the occipital cortex (adjusted mean = 14.678) in comparison to artiodactyls (adjusted mean = 10.395) (*F* = 14.05; df =13, *P* = 0.00278). When controlling for the density of cortical glia, cetaceans also had significantly higher DBH-immunoreactive bouton densities in the anterior cingulate cortex (adjusted mean = 10.62) in comparison to artiodactyls (adjusted mean = 8.01) (*F* = 9.72; df =13, *P* = 0.00889). Similar results were found for the occipital cortex, with cetaceans having significantly higher DBH-immunoreactive bouton density (adjusted mean = 14.471) compared to artiodactyls (adjusted mean = 10.395) (*F* = 11.2; df =13, *P* = 0.00581). When controlling for brain mass, cetaceans were also found to have a significantly higher DBH-immunoreactive bouton densities in the anterior cingulate (adjusted mean = 11.36) in comparison to artiodactyls (adjusted mean = 7.75) (*F* = 11.06; df = 13, *P* = 0.00604) as well as in the occipital cortex (cetacean adjusted mean = 15.406, artiodactyls adjusted mean = 10.055) (*F* = 11.85; df = 13, *P* = 0.00488).

## Supplementary Information

Supplementary Information
