# Supplementary material for: Amplification of potential thermogenetic mechanisms in cetacean brains compared to artiodactyl brains
Source: Sci Rep. 2021 Mar 9;11:5486. doi: 10.1038/s41598-021-84762-0 (PMC7970898; doi:10.1038/s41598-021-84762-0)
Supplement: Supplementary file 1 — Supplementary Information [file 41598_2021_84762_MOESM1_ESM.docx]

Supplementary Material:

Amplification of potential thermogenetic mechanisms in cetacean brains compared to artiodactyl brains

**Authors:** Paul R. Manger^1*^, Nina Patzke^1†^, Muhammad A. Spocter^1,2^, Adhil Bhagwandin^1#^, Karl Æ. Karlsson^3^, Mads F. Bertelsen^4^, Abdulaziz N. Alagaili^5^, Nigel C. Bennett^6^, Osama B. Mohammed^5^, Suzana Herculano-Houzel^7^, Patrick R. Hof^8^, Kjell Fuxe^9^.

**Affiliations**

^1^ School of Anatomical Sciences, University of the Witwatersrand, Johannesburg, South Africa.

^2^ Department of Anatomy, Des Moines University, Des Moines, IA, USA.

^3^ Biomedical Engineering, Reykjavik University, Reykjavik, Iceland.

^4^ Centre for Zoo and Wild Animal Health, Copenhagen Zoo, Frederiksberg, Denmark.

^5^ KSU Mammals Research Chair, Department of Zoology, College of Science, King Saud University, Riyadh, Saudi Arabia.

^6^ Department of Zoology and Entomology, University of Pretoria, Pretoria, South Africa.

^7^ Department of Psychology, Department of Biological Sciences, Vanderbilt Brain Institute, Vanderbilt University, Nashville, TN, USA

^8^ Nash Family Department of Neuroscience and Friedman Brain Institute, Icahn School of Medicine at Mount Sinai, New York, NY, USA.

^9^ Department of Neuroscience, Karolinska Institutet, Stockholm, Sweden.

^*^Corresponding Author: [Paul.Manger@wits.ac.za](mailto:Paul.Manger@wits.ac.za)

^†^ Current address: Institute for the Advancement of Higher Education, Hokkaido University, Sapporo, Japan.

^#^ Current address: Division of Clinical Anatomy and Biological Anthropology, Department of Human Biology, University of Cape Town, Cape Town, South Africa

Supplementary Material





Figure S1: Quantification of noradrenergic bouton density in cetartiodactyl cortical white matter. The density of dopamine-ß-hydroxylase (DBH)-immunopositive boutons in the white matter below the anterior cingulate and occipital cortex was substantially lower than that observed in the corresponding grey matter (Tables 1, S3, error bars on average bars represent one standard deviation). No statistically significant differences were noted between artiodactyls and cetaceans. Depicted is a graphical representation of the results of the stereological analysis of the density of DBH-immunopositive boutons in the white matter of the occipital and anterior cingulate cortices of the species studied. *Gm* – sand gazelle, *Gazella marica*; *Ss* – domestic pig, *Sus scrofa*; *Cn* – Nubian ibex, *Capra nubiana*; *Am* – springbok, *Antidorcas marsupialis*; *Dp* – blesbok, *Damaliscus pygargus*; *Ts* – greater kudu, *Tragelaphus strepsiceros*; *Ct* – blue wildebeest, *Connochaetes taurinus*; *Cd* – dromedary camel, *Camelus dromedarius*; *Ta* – nyala, *Tragelaphus angasii*; *Ha* – river hippopotamus, *Hippopotamus amphibius*; *Sc* – African buffalo, *Syncerus caffer*; av. – average; *Pp* – harbor porpoise, *Phocoena phocoena*; *Ba* – minke whale, *Balaenoptera acutorostrata*.


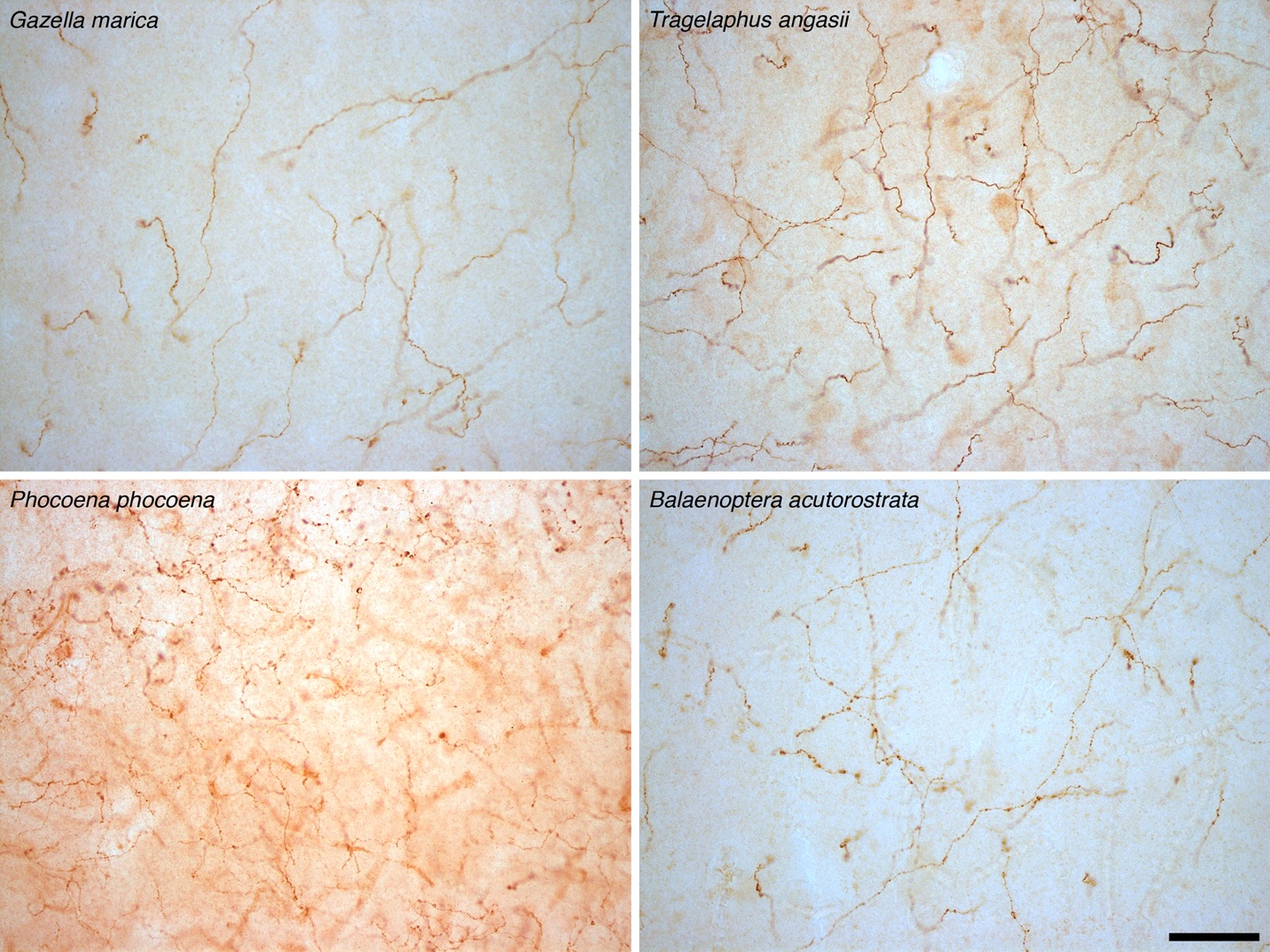


**Figure S2:** **Tyrosine hydroxylase immunoreactive boutons in cetartiodactyl cerebral cortex.** In addition to revealing the density of catecholaminergic boutons in the cerebral cortex using dopamine-ß-hydroxylase (DBH), we stained these boutons with tyrosine hydroxylase (TH), marking an earlier stage in the catecholamine biosynthetic pathway. The average density of TH-immunoreactive boutons in the cortical grey matter of the artiodactyls studied was 7778 boutons/mm^3^ (range: 4073/mm^3^ in blesbok anterior cingulate cortex to 11704/mm^3^ in river hippopotamus occipital cortex). In cetacean cortical grey matter an average density of 14599 TH-immunoreactive boutons/mm^3^ was observed (range: 10717/mm^3^ in minke whale anterior cingulate cortex to 18247/mm^3^ in harbor porpoise occipital cortex) (Tables 1, S4). Using a two-sample *t*-test we compared TH-immunoreactive bouton density in the grey matter of the anterior cingulate and occipital cortex between artiodactyls and cetaceans. Cetaceans have significantly higher mean TH-immunoreactive bouton densities in both the anterior cingulate and occipital cortex compared to artiodactyls (anterior cingulate: *t* = -6.89; df =14, *P* = 0.00137; occipital cortex: *t* = -7.22; df =14, *P* = 0.0014). In the cortical white matter an average density of 1541 TH-immunoreactive boutons/mm^3^ was observed in artiodactyls, which was significantly (anterior cingulate: *t* = 0.53; df =14, *P* = 6.02 X 10-^1^; occipital: *t* =-4.09; df =14, *P* = 0.0016) lower than, the average TH-immunoreactive bouton density found in cetacean cortical white matter (1846 boutons/mm^3^) (Fig. S4). The photomicrographs presented here depict tyrosine hydroxylase (TH) immunostained axonal boutons in the cortical grey matter of *Gazella marica*, *Tragelaphus angasii*, *Phocoena phocoena*, and *Balaenoptera acutorostrata*. The scale bar = 50 µm and applies to all photomicrographs. Note the higher density of the TH-immunoreactive boutons in the cortical grey matter of cetaceans compared to the artiodactyls (see also Fig. S3).





**Figure S3:** **Quantification of tyrosine hydroxylase immunoreactive bouton density in cetartiodactyl cerebral cortex.** Graphical representation of the results of the stereological analysis of the density of TH-immunopositive boutons in the grey matter of the occipital and anterior cingulate cortices of the species studied. Note that the density of these boutons is far higher in cetaceans than the artiodactyls (see legend of Fig. S2 for statistical results, error bars on average bars represent one standard deviation). ***Gm*** – sand gazelle, *Gazella marica*; ***Ss*** – domestic pig, *Sus scrofa*; ***Cn*** – Nubian ibex, *Capra nubiana*; ***Am*** – springbok, *Antidorcas marsupialis*; ***Dp*** – blesbok, *Damaliscus pygargus*; ***Ts*** – greater kudu, *Tragelaphus strepsiceros*; ***Ct*** – blue wildebeest, *Connochaetes taurinus*; ***Cd*** – dromedary camel, *Camelus dromedarius*; ***Ta*** – nyala, *Tragelaphus angasii*; ***Ha*** – river hippopotamus, *Hippopotamus amphibius*; ***Sc*** – African buffalo, *Syncerus caffer*; **av.** – average; ***Pp*** – harbor porpoise, *Phocoena phocoena*; ***Ba*** – minke whale, *Balaenoptera acutorostrata*.





**Figure S4:** **Quantification of tyrosine hydroxylase immunoreactive bouton density in cetartiodactyl subcortical white matter.** Graphical representation of the results of the stereological analysis of the density of TH-immunopositive boutons in the white matter of the occipital and anterior cingulate cortices of the species studied. Note that the density of these boutons does not vary significantly across the species studied, although the average for cetaceans is slightly higher than that seen in the artiodactyls (see legend of Fig. S2 for statistical results, error bars on average bars represent one standard deviation). ***Gm*** – sand gazelle, *Gazella marica*; ***Ss*** – domestic pig, *Sus scrofa*; ***Cn*** – Nubian ibex, *Capra nubiana*; ***Am*** – springbok, *Antidorcas marsupialis*; ***Dp*** – blesbok, *Damaliscus pygargus*; ***Ts*** – greater kudu, *Tragelaphus strepsiceros*; ***Ct*** – blue wildebeest, *Connochaetes taurinus*; ***Cd*** – dromedary camel, *Camelus dromedarius*; ***Ta*** – nyala, *Tragelaphus angasii*; ***Ha*** – river hippopotamus, *Hippopotamus amphibius*; ***Sc*** – African buffalo, *Syncerus caffer*; **av.** – average; ***Pp*** – harbor porpoise, *Phocoena phocoena*; ***Ba*** – minke whale, *Balaenoptera acutorostrata*.


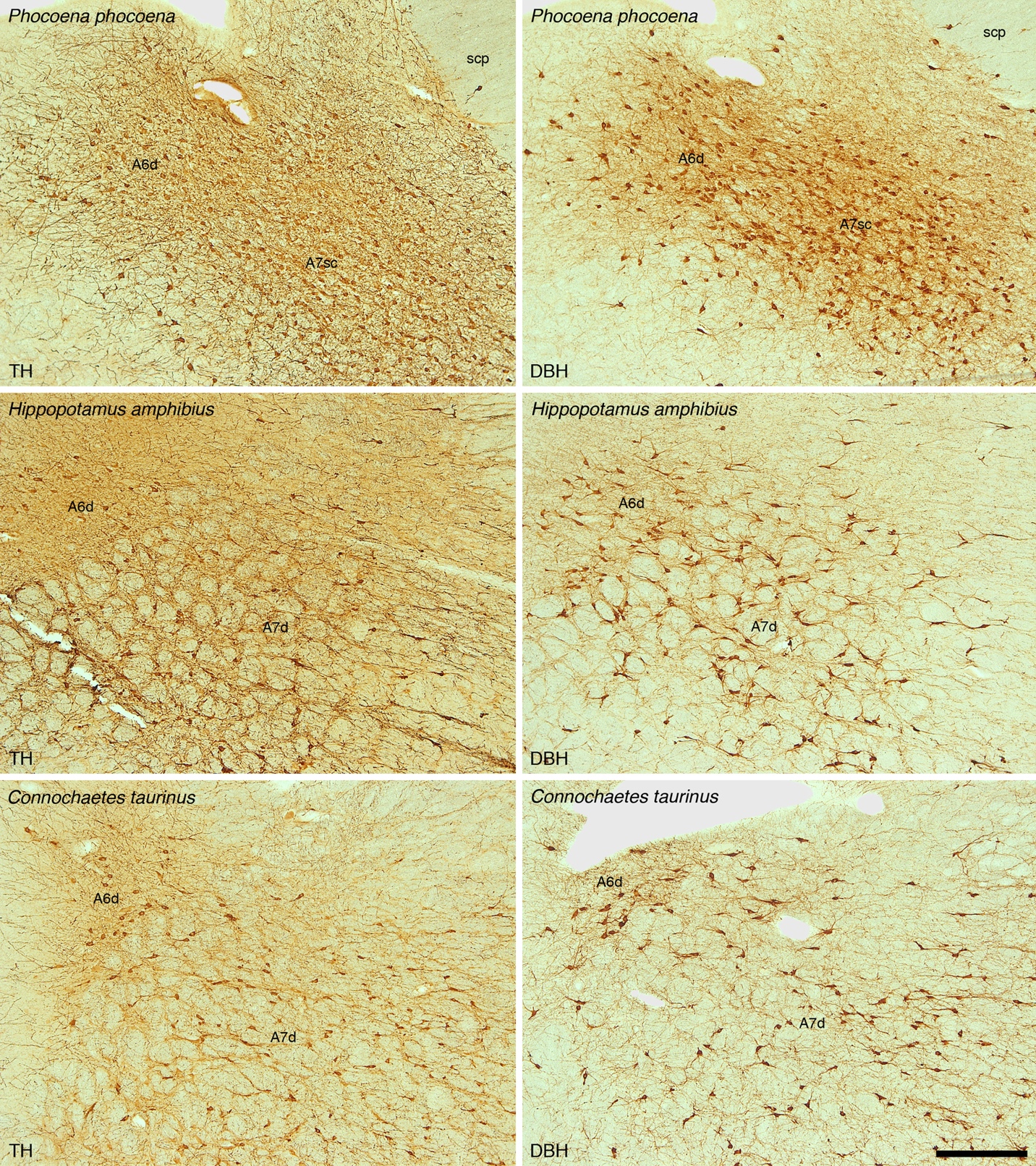


**Figure S5: The locus coeruleus of cetartiodactyls.** To support the concept that the noradrenergic innervation of the cerebral cortex arises from the locus coeruleus complex in the species studied, we examined the locus coeruleus with antibodies to tyrosine hydroxylase (TH) and dopamine-ß-hydroxylase (DBH). In all cases, the pattern of immunostaining indicates that the locus coeruleus of cetartiodactyls is the origin of noradrenergic projections throughout the brain. The photomicrographs provided here depict coronal sections through the locus coeruleus complex of the harbor porpoise (*Phocoena phocoena*), river hippopotamus (*Hippopotamus amphibius)* and blue wildebeest (*Connochaetes taurinus*) immunostained for TH (left column) and DBH (right column). Scale bar = 500 µm and applies to all. In all images dorsal is to the top and medial to the left. **A6d** – diffuse portion of locus coeruleus, **A7d** – diffuse portion of nucleus subcoeruleus, **A7sc** – compact portion of nucleus subcoeruleus.





**Figure S6: Unedited full-length Western immunoblots.** Edited and cropped versions of these immunoblots are presented in Figures 2 (**A**) and 4 (**B** and **C**) of the current manuscript. (**A**) This unedited full-length Western immunoblot, placed in Figure 2, shows the specificity of the UCP1 antibody used in the current study to brown fat taken from a laboratory rat. (**B** and **C**) These unedited full-length Western immunoblot, placed in Figure 4, show the specificity of the UCP4 antibody used in the current study to cerebral cortical tissue from a range of cetartiodactyl species, as well as the laboratory rat. The unedited full-length Western immunoblots underwent cropping and basic editing (for contrast, brightness and levels) for use in the Figures presented in the main manuscript. Details of the species corresponding to each column, and the molecular weights of the control bands, are presented in Figures 2 and 4.

**Table S1** Stereological parameters used in the estimation of UCP1-immunostained neuronal densities in the grey matter of the anterior cingulate (**AC**) and occipital (**OC**) cortices in the species studied.

| Species | Counting Frame Area (µm²) | Sampling Grid Area (µm²) | Disector height (µm) | Section cut thickness (µm) | Measured mounted thickness (µm) | Guard zone (µm) | Section interval | Number of sections | Number of sampling sites |
| --- | --- | --- | --- | --- | --- | --- | --- | --- | --- |
| Sand gazelle (AC) | 6400 | 1102500 | 4 | 50 | 13.4 | 2 | 1 | 3 | 40 |
| Sand gazelle (OC) | 6400 | 1102500 | 4 | 50 | 16.7 | 2 | 1 | 3 | 42 |
| Domestic pig (AC) | 6400 | 1102500 | 4 | 50 | 14.2 | 2 | 1 | 3 | 88 |
| Domestic pig (OC) | 6400 | 1102500 | 4 | 50 | 15.8 | 2 | 1 | 3 | 81 |
| Nubian ibex (AC) | 6400 | 1102500 | 4 | 50 | 14.6 | 2 | 1 | 3 | 109 |
| Nubian ibex (OC) | 6400 | 1102500 | 4 | 50 | 15.6 | 2 | 1 | 3 | 104 |
| Springbok (AC) | 6400 | 1102500 | 4 | 50 | 9.9 | 2 | 1 | 3 | 96 |
| Springbok (OC) | 6400 | 1102500 | 4 | 50 | 9.3 | 2 | 1 | 3 | 86 |
| Blesbok (AC) | 6400 | 1102500 | 4 | 50 | 11.4 | 2 | 1 | 3 | 139 |
| Blesbok (OC) | 6400 | 1102500 | 4 | 50 | 10.7 | 2 | 1 | 3 | 115 |
| Greater kudu (AC) | 6400 | 1102500 | 4 | 50 | 8.5 | 2 | 1 | 3 | 108 |
| Greater kudu (OC) | 6400 | 1102500 | 4 | 50 | 9.3 | 2 | 1 | 3 | 148 |
| Blue wildebeest (AC) | 6400 | 1102500 | 4 | 50 | 10.2 | 2 | 1 | 3 | 158 |
| Blue wildebeest (OC) | 6400 | 1102500 | 4 | 50 | 10 | 2 | 1 | 3 | 115 |
| Dromedary camel (AC) | 6400 | 1102500 | 4 | 50 | 16.8 | 2 | 1 | 3 | 54 |
| Dromedary camel (OC) | 6400 | 1102500 | 4 | 50 | 12.2 | 2 | 1 | 3 | 52 |
| Nyala (AC) | 6400 | 1102500 | 4 | 50 | 10.2 | 2 | 1 | 3 | 59 |
| Nyala (OC) | 6400 | 1102500 | 4 | 50 | 11.2 | 2 | 1 | 3 | 94 |
| River hippopotamus (AC) | 6400 | 1102500 | 4 | 50 | 8.6 | 2 | 1 | 3 | 129 |
| River hippopotamus (OC) | 6400 | 1102500 | 4 | 50 | 9.6 | 2 | 1 | 3 | 111 |
| African buffalo (AC) | 6400 | 1102500 | 4 | 50 | 12.9 | 2 | 1 | 3 | 108 |
| African buffalo (OC) | 6400 | 1102500 | 4 | 50 | 13.1 | 2 | 1 | 3 | 94 |
| Harbor porpoise 1 (AC) | 6400 | 1102500 | 4 | 50 | 10.6 | 2 | 1 | 3 | 100 |
| Harbor porpoise 1 (OC) | 6400 | 1102500 | 4 | 50 | 13.9 | 2 | 1 | 3 | 124 |
| Harbor porpoise 2 (AC) | 6400 | 1102500 | 4 | 50 | 11.2 | 2 | 1 | 3 | 112 |
| Harbor porpoise 2 (OC) | 6400 | 1102500 | 4 | 50 | 12.8 | 2 | 1 | 3 | 130 |
| Minke whale 1 (AC) | 6400 | 1102500 | 4 | 50 | 12.4 | 2 | 1 | 3 | 163 |
| Minke whale 1 (OC) | 6400 | 1102500 | 4 | 50 | 13.8 | 2 | 1 | 3 | 226 |
| Minke whale 2 (AC) | 6400 | 1102500 | 4 | 50 | 12.4 | 2 | 1 | 3 | 112 |
| Minke whale 2 (OC) | 6400 | 1102500 | 4 | 50 | 13.9 | 2 | 1 | 3 | 168 |

**Table S2:** Stereological parameters used in the estimation of UCP4-immunostained glia densities in the grey and white matter of the anterior cingulate (**AC**) and occipital (**OC**) cortices in the cetacean species studied.

| Species | Counting Frame Area (µm²) | Sampling Grid Area (µm²) | Counting Frame Area (µm²) | Sampling Grid Area (µm²) | Disector height (µm) | Section cut thickness (µm) | Measured mounted thickness (µm) | Guard zone (µm) | Section interval | Number of sections | Number of sampling sites |
| --- | --- | --- | --- | --- | --- | --- | --- | --- | --- | --- | --- |
|  | Grey matter | | White matter | |  |  |  |  |  |  |  |
| Harbor porpoise 1 (AC) | 6400 | 1102500 | 6400 | 1102500 | 4 | 50 | 18.5 | 2 | 1 | 3 | 85 |
| Harbor porpoise 1 (OC) | 6400 | 1102500 | 6400 | 1102500 | 4 | 50 | 20.3 | 2 | 1 | 3 | 69 |
| Harbor porpoise 2 (AC) | 6400 | 1102500 | 6400 | 1102500 | 4 | 50 | 15.9 | 2 | 1 | 3 | 88 |
| Harbor porpoise 2 (OC) | 6400 | 1102500 | 6400 | 1102500 | 4 | 50 | 12.6 | 2 | 1 | 3 | 85 |
| Minke whale 1 (AC) | 6400 | 1102500 | 6400 | 1102500 | 4 | 50 | 21.8 | 2 | 1 | 3 | 105 |
| Minke whale 1 (OC) | 6400 | 1102500 | 6400 | 1102500 | 4 | 50 | 17.5 | 2 | 1 | 3 | 180 |
| Minke whale 2 (AC) | 6400 | 1102500 | 6400 | 1102500 | 4 | 50 | 14.7 | 2 | 1 | 3 | 178 |
| Minke whale 2 (OC) | 6400 | 1102500 | 6400 | 1102500 | 4 | 50 | 18.5 | 2 | 1 | 3 | 181 |

**Table S3:** Stereological parameters used in the estimation of dopamine-ß-hydroxylase (DBH)-immunoreactive bouton densities in the grey and white matter of the anterior cingulate (**AC**) and occipital (**OC**) cortices in the species studied.

| Species | Counting frame size (µm) | Sampling grid size (µm) | Counting frame size (µm) | Sampling grid size (µm) | Disector height (µm) | Section cut thickness (µm) | Measured mounted thickness (µm) | Guard zone (µm) | Section interval | Number of sections | Number of sampling sites |
| --- | --- | --- | --- | --- | --- | --- | --- | --- | --- | --- | --- |
|  | Grey matter | | White matter | |  |  |  |  |  |  |  |
| Sand gazelle (AC) | 100 x 100 | 200 x 200 | 100 x 100 | 200 x 200 | 17 | 50 | 21.4 | 2 | 1 | 3 | 114 |
| Sand gazelle (OC) | 100 x 100 | 200 x 200 | 100 x 100 | 200 x 200 | 17 | 50 | 22.3 | 2 | 1 | 3 | 112 |
| Domestic pig (AC) | 100 x 100 | 200 x 200 | 100 x 100 | 200 x 200 | 16 | 50 | 20.5 | 2 | 1 | 3 | 118 |
| Domestic pig (OC) | 100 x 100 | 200 x 200 | 100 x 100 | 200 x 200 | 16 | 50 | 20.3 | 2 | 1 | 3 | 116 |
| Nubian ibex (AC) | 100 x 100 | 200 x 200 | 100 x 100 | 200 x 200 | 18 | 50 | 23.2 | 2 | 1 | 3 | 116 |
| Nubian ibex (OC) | 100 x 100 | 200 x 200 | 100 x 100 | 200 x 200 | 18 | 50 | 22.4 | 2 | 1 | 3 | 119 |
| Springbok (AC) | 100 x 100 | 200 x 200 | 100 x 100 | 200 x 200 | 18 | 50 | 23.5 | 2 | 1 | 3 | 88 |
| Springbok (OC) | 100 x 100 | 200 x 200 | 100 x 100 | 200 x 200 | 18 | 50 | 22.7 | 2 | 1 | 3 | 91 |
| Blesbok (AC) | 100 x 100 | 200 x 200 | 100 x 100 | 200 x 200 | 19 | 50 | 23.7 | 2 | 1 | 3 | 107 |
| Blesbok (OC) | 100 x 100 | 200 x 200 | 100 x 100 | 200 x 200 | 19 | 50 | 24.0 | 2 | 1 | 3 | 93 |
| Greater kudu (AC) | 100 x 100 | 250 x 250 | 100 x 100 | 250 x 250 | 17 | 50 | 22.1 | 2 | 1 | 3 | 116 |
| Greater kudu (OC) | 100 x 100 | 250 x 250 | 100 x 100 | 250 x 250 | 17 | 50 | 21.8 | 2 | 1 | 3 | 107 |
| Blue wildebeest (AC) | 100 x 100 | 250 x 250 | 100 x 100 | 250 x 250 | 20 | 50 | 24.5 | 2 | 1 | 3 | 114 |
| Blue wildebeest (OC) | 100 x 100 | 250 x 250 | 100 x 100 | 250 x 250 | 20 | 50 | 24.6 | 2 | 1 | 3 | 101 |
| Dromedary camel (AC) | 100 x 100 | 250 x 250 | 100 x 100 | 250 x 250 | 18 | 50 | 23.7 | 2 | 1 | 3 | 119 |
| Dromedary camel (OC) | 100 x 100 | 250 x 250 | 100 x 100 | 250 x 250 | 18 | 50 | 22.8 | 2 | 1 | 3 | 107 |
| Nyala (AC) | 100 x 100 | 200 x 200 | 100 x 100 | 200 x 200 | 19 | 50 | 23.8 | 2 | 1 | 3 | 114 |
| Nyala (OC) | 100 x 100 | 200 x 200 | 100 x 100 | 200 x 200 | 19 | 50 | 24.3 | 2 | 1 | 3 | 107 |
| River hippopotamus (AC) | 100 x 100 | 250 x 250 | 100 x 100 | 250 x 250 | 18 | 50 | 23.1 | 2 | 1 | 3 | 96 |
| River hippopotamus (OC) | 100 x 100 | 250 x 250 | 100 x 100 | 250 x 250 | 18 | 50 | 22.4 | 2 | 1 | 3 | 102 |
| African buffalo (AC) | 100 x 100 | 250 x 250 | 100 x 100 | 250 x 250 | 18 | 50 | 22.8 | 2 | 1 | 3 | 113 |
| African buffalo (OC) | 100 x 100 | 250 x 250 | 100 x 100 | 250 x 250 | 18 | 50 | 22.5 | 2 | 1 | 3 | 84 |
| Harbor porpoise 1 (AC) | 100 x 100 | 250 x 250 | 100 x 100 | 250 x 250 | 19 | 50 | 23.7 | 2 | 1 | 3 | 113 |
| Harbor porpoise 1 (OC) | 100 x 100 | 250 x 250 | 100 x 100 | 250 x 250 | 19 | 50 | 23.2 | 2 | 1 | 3 | 99 |
| Harbor porpoise 2 (AC) | 100 x 100 | 250 x 250 | 100 x 100 | 250 x 250 | 16 | 50 | 20.4 | 2 | 1 | 3 | 97 |
| Harbor porpoise 2 (OC) | 100 x 100 | 250 x 250 | 100 x 100 | 250 x 250 | 16 | 50 | 20.5 | 2 | 1 | 3 | 113 |
| Minke whale 1 (AC) | 100 x 100 | 250 x 250 | 100 x 100 | 250 x 250 | 16 | 50 | 21.3 | 2 | 1 | 3 | 118 |
| Minke whale 1 (OC) | 100 x 100 | 250 x 250 | 100 x 100 | 250 x 250 | 16 | 50 | 20.8 | 2 | 1 | 3 | 121 |
| Minke whale 2 (AC) | 100 x 100 | 250 x 250 | 100 x 100 | 250 x 250 | 16 | 50 | 21.5 | 2 | 1 | 3 | 79 |
| Minke whale 2 (OC) | 100 x 100 | 250 x 250 | 100 x 100 | 250 x 250 | 16 | 50 | 20.6 | 2 | 1 | 3 | 121 |

**Table S4:** Stereological parameters used in the estimation of tyrosine hydroxylase (TH)-immunoreactive bouton densities in the grey and white matter of the anterior cingulate (**AC**) and occipital (**OC**) cortices in the species studied.

| Species | Counting frame size (µm) | Sampling grid size (µm) | Counting frame size (µm) | Sampling grid size (µm) | Disector height (µm) | Section cut thickness (µm) | Measured mounted thickness (µm) | Guard zone (µm) | Section interval | Number of sections | Number of sampling sites |
| --- | --- | --- | --- | --- | --- | --- | --- | --- | --- | --- | --- |
|  | Grey matter | | White matter | |  |  |  |  |  |  |  |
| Sand gazelle (AC) | 100 x 100 | 200 x 200 | 100 x 100 | 200 x 200 | 16 | 50 | 20.8 | 2 | 1 | 3 | 116 |
| Sand gazelle (OC) | 100 x 100 | 200 x 200 | 100 x 100 | 200 x 200 | 16 | 50 | 20.9 | 2 | 1 | 3 | 112 |
| Domestic pig (AC) | 100 x 100 | 200 x 200 | 100 x 100 | 200 x 200 | 16 | 50 | 21.2 | 2 | 1 | 3 | 113 |
| Domestic pig (OC) | 100 x 100 | 200 x 200 | 100 x 100 | 200 x 200 | 16 | 50 | 21.2 | 2 | 1 | 3 | 116 |
| Nubian ibex (AC) | 100 x 100 | 200 x 200 | 100 x 100 | 200 x 200 | 19 | 50 | 24 | 2 | 1 | 3 | 114 |
| Nubian ibex (OC) | 100 x 100 | 200 x 200 | 100 x 100 | 200 x 200 | 19 | 50 | 23.2 | 2 | 1 | 3 | 115 |
| Springbok (AC) | 100 x 100 | 200 x 200 | 100 x 100 | 200 x 200 | 16 | 50 | 21.8 | 2 | 1 | 3 | 89 |
| Springbok (OC) | 100 x 100 | 200 x 200 | 100 x 100 | 200 x 200 | 16 | 50 | 21.6 | 2 | 1 | 3 | 97 |
| Blesbok (AC) | 100 x 100 | 200 x 200 | 100 x 100 | 200 x 200 | 18 | 50 | 23.4 | 2 | 1 | 3 | 102 |
| Blesbok (OC) | 100 x 100 | 200 x 200 | 100 x 100 | 200 x 200 | 18 | 50 | 22.5 | 2 | 1 | 3 | 97 |
| Greater kudu (AC) | 100 x 100 | 250 x 250 | 100 x 100 | 250 x 250 | 17 | 50 | 21.8 | 2 | 1 | 3 | 117 |
| Greater kudu (OC) | 100 x 100 | 250 x 250 | 100 x 100 | 250 x 250 | 17 | 50 | 22.5 | 2 | 1 | 3 | 109 |
| Blue wildebeest (AC) | 100 x 100 | 250 x 250 | 100 x 100 | 250 x 250 | 17 | 50 | 21.9 | 2 | 1 | 3 | 114 |
| Blue wildebeest (OC) | 100 x 100 | 250 x 250 | 100 x 100 | 250 x 250 | 17 | 50 | 21.7 | 2 | 1 | 3 | 110 |
| Dromedary camel (AC) | 100 x 100 | 250 x 250 | 100 x 100 | 250 x 250 | 18 | 50 | 22.6 | 2 | 1 | 3 | 114 |
| Dromedary camel (OC) | 100 x 100 | 250 x 250 | 100 x 100 | 250 x 250 | 18 | 50 | 22.2 | 2 | 1 | 3 | 113 |
| Nyala (AC) | 100 x 100 | 200 x 200 | 100 x 100 | 200 x 200 | 17 | 50 | 22.4 | 2 | 1 | 3 | 116 |
| Nyala (OC) | 100 x 100 | 200 x 200 | 100 x 100 | 200 x 200 | 17 | 50 | 21.8 | 2 | 1 | 3 | 113 |
| River hippopotamus (AC) | 100 x 100 | 250 x 250 | 100 x 100 | 250 x 250 | 17 | 50 | 22.4 | 2 | 1 | 3 | 95 |
| River hippopotamus (OC) | 100 x 100 | 250 x 250 | 100 x 100 | 250 x 250 | 17 | 50 | 22.6 | 2 | 1 | 3 | 97 |
| African buffalo (AC) | 100 x 100 | 250 x 250 | 100 x 100 | 250 x 250 | 18 | 50 | 22.7 | 2 | 1 | 3 | 112 |
| African buffalo (OC) | 100 x 100 | 250 x 250 | 100 x 100 | 250 x 250 | 18 | 50 | 22.3 | 2 | 1 | 3 | 104 |
| Harbor porpoise 1 (AC) | 100 x 100 | 250 x 250 | 100 x 100 | 250 x 250 | 17 | 50 | 22.1 | 2 | 1 | 3 | 114 |
| Harbor porpoise 1 (OC) | 100 x 100 | 250 x 250 | 100 x 100 | 250 x 250 | 17 | 50 | 21.2 | 2 | 1 | 3 | 117 |
| Harbor porpoise 2 (AC) | 100 x 100 | 250 x 250 | 100 x 100 | 250 x 250 | 18 | 50 | 22.7 | 2 | 1 | 3 | 113 |
| Harbor porpoise 2 (OC) | 100 x 100 | 250 x 250 | 100 x 100 | 250 x 250 | 18 | 50 | 23.2 | 2 | 1 | 3 | 90 |
| Minke whale 1 (AC) | 100 x 100 | 250 x 250 | 100 x 100 | 250 x 250 | 18 | 50 | 23 | 2 | 1 | 3 | 118 |
| Minke whale 1 (OC) | 100 x 100 | 250 x 250 | 100 x 100 | 250 x 250 | 18 | 50 | 23.2 | 2 | 1 | 3 | 118 |
| Minke whale 2 (AC) | 100 x 100 | 250 x 250 | 100 x 100 | 250 x 250 | 18 | 50 | 23.3 | 2 | 1 | 3 | 118 |
| Minke whale 2 (OC) | 100 x 100 | 250 x 250 | 100 x 100 | 250 x 250 | 18 | 50 | 22.7 | 2 | 1 | 3 | 114 |
